# Resolution of intussusception after spontaneous expulsion of an ileal lipoma per rectum: a case report and literature review

**DOI:** 10.1186/1477-7819-12-143

**Published:** 2014-05-07

**Authors:** Bin Kang, Qingkai Zhang, Dong Shang, Qingqiang Ni, Faheem Muhammad, Li Hou, Wenjun Cui

**Affiliations:** 1 Department of Acute Abdominal Surgery, First Affiliated Hospital, Dalian Medical University, No. 222 Zhongshan Road, Dalian 116011, Liaoning Province, China; 2 Department of Pathology, Dalian Medical University, No. 9 Lvshun South Road, Dalian 116044, Liaoning Province, China; 3 Department of Radiology, First Affiliated Hospital, Dalian Medical University, No. 222 Zhongshan Road, Dalian 116011, Liaoning Province, China

**Keywords:** Expulse, Intussusception, Lipoma, Self-healing, Small intestine

## Abstract

We herein report a case of spontaneous rectal expulsion of an ileal lipoma in a 65-year-old female patient who presented with recurrent attacks of subacute intestinal obstruction. During each episode, the patient developed severe abdominal pain and expelled a fleshy mass from her rectum. The fleshy mass was histopathologically diagnosed as a lipoma comprising fat cells, fibers, and blood vessels. Upon expulsion, the pain disappeared and the intussusception was immediately resolved. Colonoscopic examination revealed a 2.5-cm diameter ulcerated lesion near the ileocecal valve, which was confirmed to be inflammation by pathological examination. A subsequent barium series revealed a normal colonic tract, and the patient remained completely symptom-free for 4 months after the incident. According to the relevant literature and our clinical experience, the treatment method for a lipoma depends on the patient’s clinical manifestations and the size of the tumor. However, the various diagnostic and therapeutic modalities currently available continue to be debated; whether an asymptomatic lipoma requires treatment is controversial. When histopathological examination results allow for the exclusion of malignant lesions such as sarcoma, a lipoma can be resected surgically.

## Background

Small intestinal tumors are extremely rare, accounting for only 1% to 2% of gastrointestinal tumors worldwide [1], and only around 30% of small intestinal tumors are benign. Lipoma, which is less prevalent than leiomyoma and adenoma, is the third most common primary benign tumor in the gut [2]. Small bowel tumors are rare entities that often present with nonspecific symptoms. When these tumors are >2 cm in diameter, abdominal pain, hematochezia, and/or incomplete intestinal obstruction may appear. The lack of specific clinical manifestations makes it difficult to reach a definite preoperative diagnosis; sometimes the lipoma is even ignored after the development of an intussusception. Moreover, it is difficult to identify symptomatic patients with malignant tumors, which can consequently be misdiagnosed. Most intestinal lipomas are located in the distal ileum and colorectal region (mainly the right colon) and are rarely located in the stomach or proximal small intestine. Intestinal lipomas seldom deteriorate, and they do not relapse after cure.

Intussusception was reported for the first time in 1674 by Barbette of Amsterdam [3]. The development of intussusception in adults is extremely rare and has a variety of etiologies. Neoplasia is the most common cause and is present in approximately 65% of adult intussusception cases [4]. Adult patients with intussusception may or may not be symptomatic, and symptoms can be acute, intermittent, or chronic. Therefore, intussusception is difficult to diagnose. The manifestations of symptomatic lipoma include abdominal pain, hemorrhage, or incomplete intestinal obstruction. Because of their intramural location, lipomas can also serve as the leading point for intussusceptions. A correct and timely diagnosis is important to avoid complications such as bowel infarction and perforation secondary to obstruction.

## Case presentation

**Figure 1:**
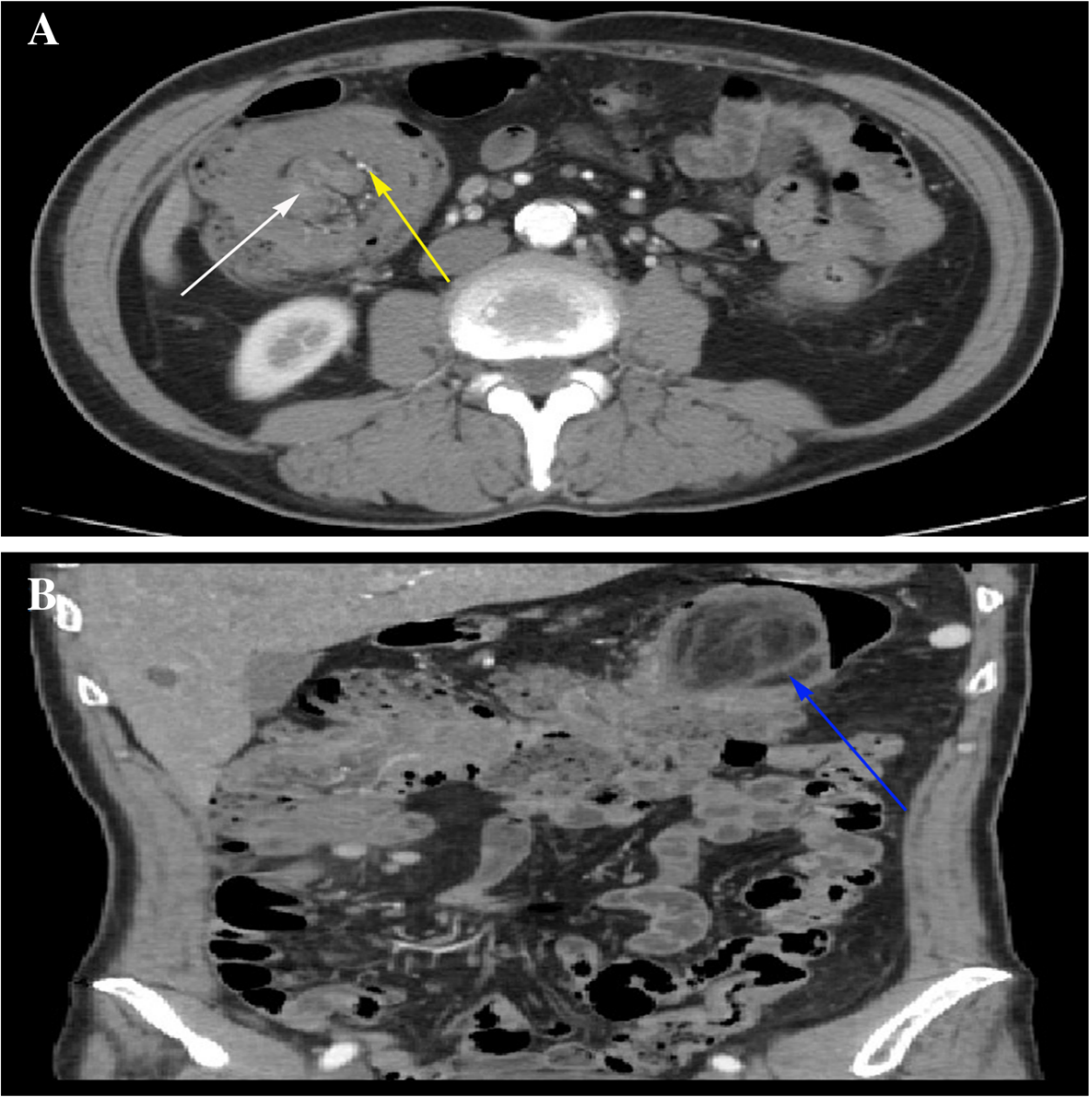
**Transverse and coronary contrast**-**enhanced computed tomography (CECT) of the abdomen. (A)** Transverse CECT showed the bowel intussusception (white arrow) entered the intestinal lumen on the right middle abdomen. Enhanced blood vessel (yellow arrow) also entered the intestinal lumen along with the bowel. **(B)** Coronary CECT showed that the tumor was located at the splenic flexure of the colon (blue arrow) and was accompanied by local intestinal expansion.

A 65-year-old woman was admitted to the emergency department with a 3-year history of intermittent abdominal pain that was exacerbated and accompanied by nausea for the past 5 days. The pain was moderate, paroxysmal, and colicky in nature; it was present mainly in the right lower quadrant and radiated to the back. She had no fever and reported intermittent defecation without nausea or vomiting. The patient had previously been seen at another hospital, where abdominal ultrasound and computed tomography (CT) examination revealed either a smooth, well-circumscribed mass within the lumen of the bowel or an intussusception. Her symptoms improved after conservative treatment. Physical examination revealed tenderness in the right lower quadrant and a smooth, well-circumscribed mass of approximately 7.0 × 5.0 cm was palpated in the epigastrium. Abdominal CT revealed a small bowel intussusception in the right epigastric region (Figure 1A and B).

The patient had no history of surgery, trauma, or other diseases. Laboratory test results showed a white blood cell count of 7.80 × 10^9^/L, a neutrophil level of 71.3%, and a hemoglobin level of 104 g/L. Other examination results were normal. The preliminary diagnosis was considered to be an intestinal tumor with intussusception.

**Figure 2:**
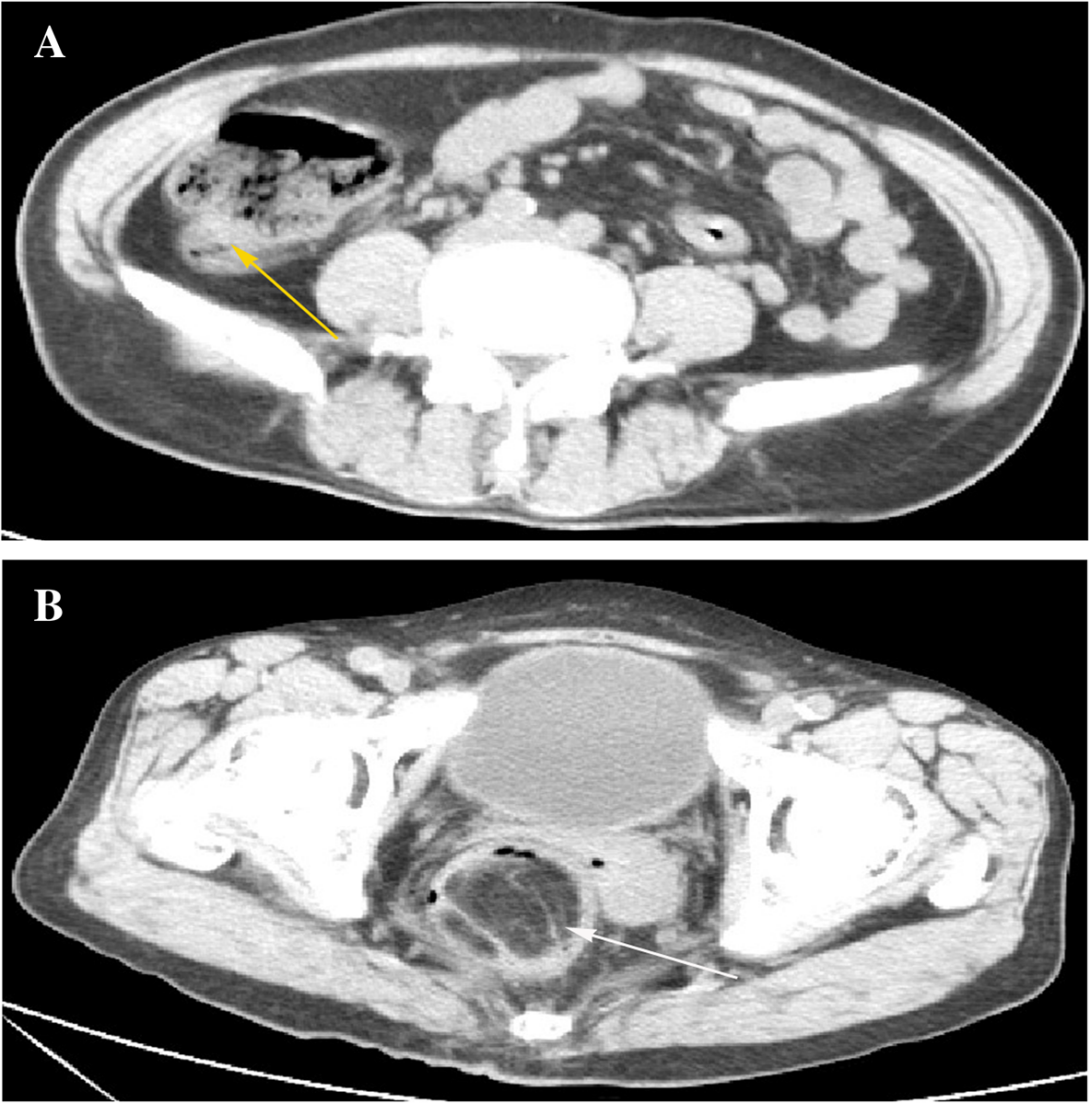
**Computed tomography (CT) of the abdomen. (A)** CT showed a mass shadow in the ileocecal valve with a maximum size of 2.90 × 3.22 cm (yellow arrow). **(B)** CT showed the rectum expanded with fat density and space inside a shadow (white arrow).

**Figure 3:**
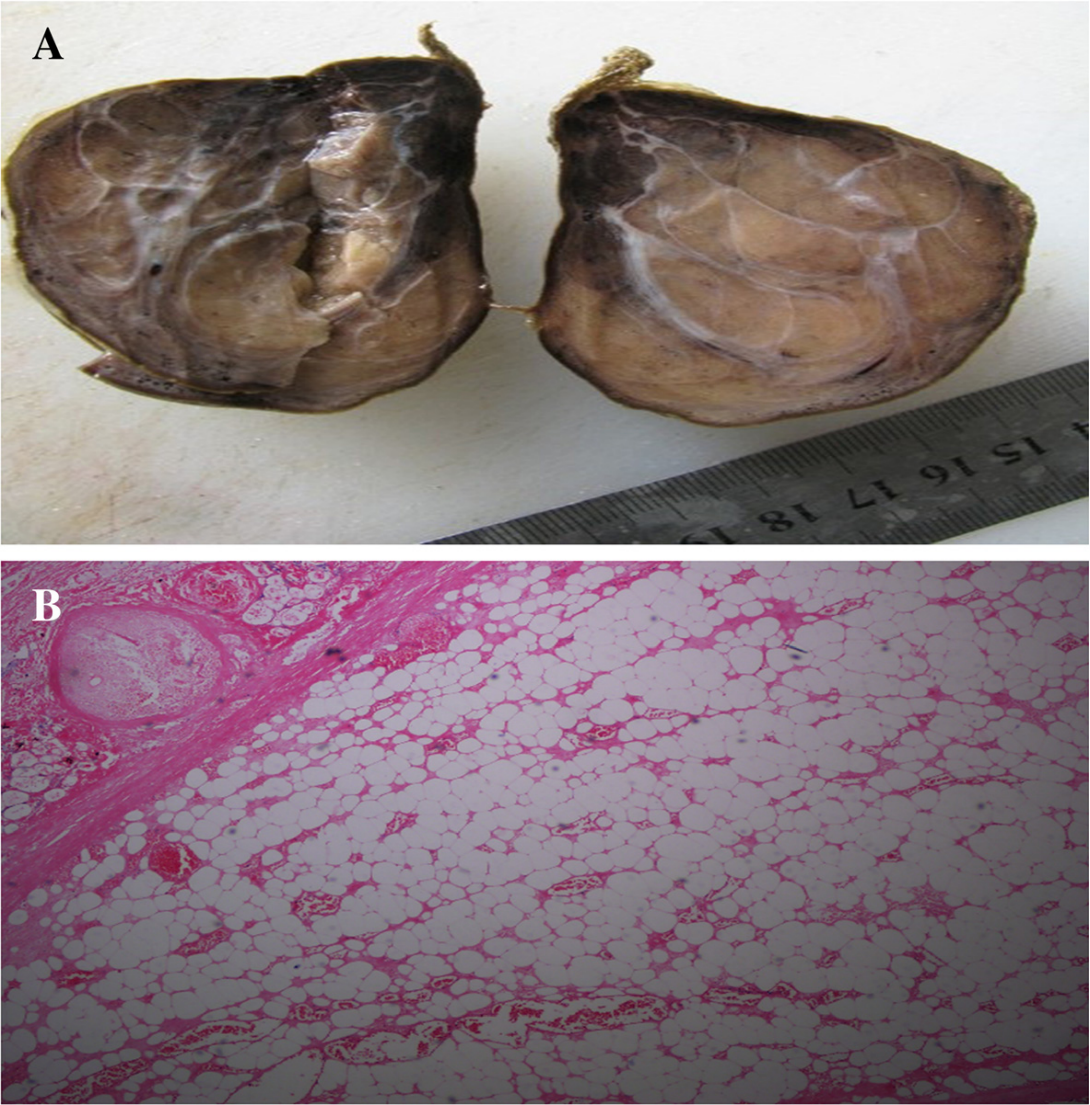
**Tumor and histopathological features. (A)** A mass section showed a gray surface and fine texture by formalin infiltration. **(B)** Microscopic examination showed fat cells, blood vessels, and fiber cells arranged in a leaf pattern, with no heterogeneous nucleus or seedless division in the submucosal layer (hematoxylin and eosin staining, ×40).

After admission, the patient was treated with oral therapy for bowel lubrication and experienced gradual relief of her abdominal pain. A vegetative mass was then extruded from the rectum when the patient defecated. However, it could not be completely discharged. Digital rectal examination revealed a well-circumscribed neoplasm with poor mobility in the center of the rectal lumen. A small amount of blood was present on the glove after digital examination. Abdominal CT showed a mass shadow in the ileocecal valve region with a maximum size of approximately 2.90 × 3.22 cm (Figure 2A) and an expanded rectum with fat density and space within a shadow (Figure 2B). We actively enlarged the anus and administered a strong oral laxative. The patient then successfully defecated the mass. The mass was approximately 7.0 × 4.5 × 3.6 cm in size and of medium hardness; it had a smooth gray surface and a fine texture (Figure 3A). Microscopic examination showed fat cells, blood vessels, and fiber cells arranged in a leaf pattern, with no heterogeneous nucleus or seedless division in the submucosal layer. The pathologic diagnosis was a bleeding submucosal lipoma with intestinal mucosal necrosis (Figure 3B).

**Figure 4:**
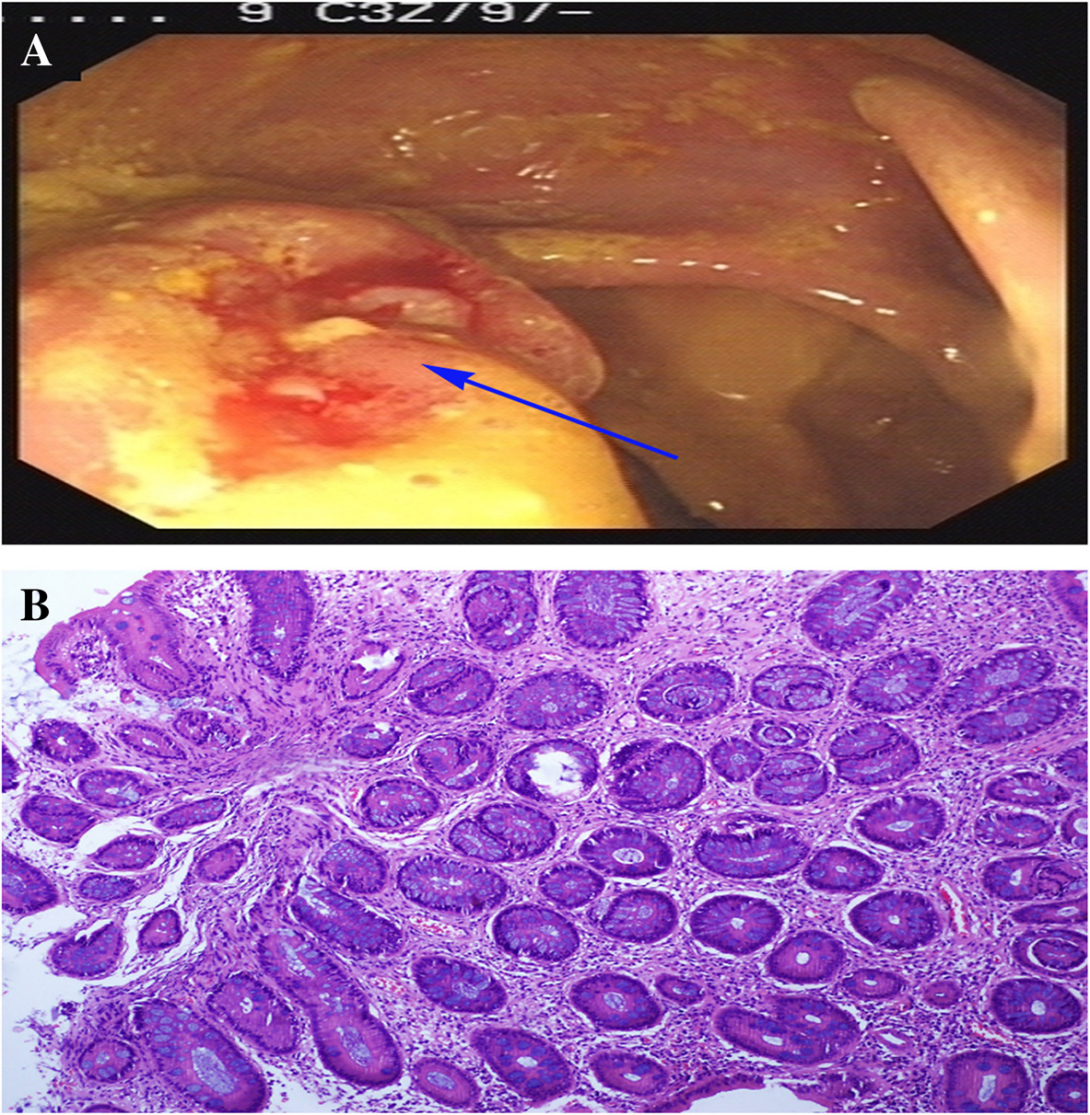
**Colonoscopy and histopathological features. (A)** Colonoscopy showed an approximately 2.5-cm diameter ulcerative lesion (blue arrow) near the ileocecal valve, surrounded by mucosal congestion. **(B)** Microscopic examination showed regularly arranged glands and epithelia with no atypia, but interstitial edema, eosinophils, and lymphocyte infiltration were present (hematoxylin and eosin staining, ×100).

After expulsion of the mass, the abdominal pain was completely relieved. Follow-up abdominal CT showed that the mass in the splenic flexure of the colon had disappeared and that the intussusception had been resolved. Colonoscopy showed an ulcerative lesion approximately 2.5 cm in diameter near the ileocecal valve that was surrounded by mucosal congestion (Figure 4A). Microscopic examination revealed regularly arranged glands; the epithelia were not atypical, but interstitial edema, eosinophils, and lymphocyte infiltration were present (Figure 4B). The pathologic diagnosis was inflammation of the ileocecal valve. Four months later, colonoscopy indicated that the inflammation of the ileocecal valve had healed and that the mucosa was intact. Subsequent capsule endoscopy and enteroscopy examination demonstrated no pathological changes.

## Discussion

When the lipoma moved to the splenic flexure, as in this case, abdominal CT still showed signs of intussusception, indicating that the mass was connected to a pedicle. The long duration of the intussusception, the gravity of the lipoma, and the presence of intestinal peristalsis gave rise to ischemia, necrosis, and breakage of the pedicle. Thus, the lipoma fell from the descending colon to the rectum, and the intussusception was resolved. After anal dilatation and catharsis treatment, the mass was spontaneously expelled. Abdominal CT and colonoscopy showed the mass shadow with an ulcerative lesion near the ileocecal valve as well as congestion of the surrounding mucosa. We consider that the mass shadow was caused by intestinal edema and thickening. The ulcerative lesion may have been caused by the impression of the mass when it fell to the ileocecal valve.

An intestinal lipoma is a benign tumor that can appear in any part of the gut. The terminal ileum is the most common location in the small intestine. There are three pathological types of intestinal lipomas. The submucosal type is the most common, accounting for more than 90% of intestinal lipomas, and grows within the submucosal layer, protruding into the lumen. The intermuscular type is located within the muscular layer. The subserosal type, which is asymptomatic, grows within the subserosal layer and protrudes out of the gut. Larger lipomas can be palpated as a smooth, movable abdominal lump. The tumor in our case was a submucosal lipoma. The patient spontaneously expelled the lipoma from her rectum before the development of intestinal ischemia and necrosis, and the intussusception was resolved spontaneously.

Imaging examination and colonoscopy contribute to the preoperative diagnosis of intestinal lipomas. Intestinal lipomas can be diagnosed by conventional endoscopy, capsule endoscopy, and barium examination. The presence of a round filling defect with or without a pedicle in the lumen on barium enema examination or small intestinal barium enema intubation examination indicates a lipoma. Ultrasound is usually the first diagnostic modality chosen, especially when the lipoma is accompanied by intestinal intussusception. The characteristic ultrasound finding associated with an intussusception is the “target sign”, in which the outermost layer represents the sheath, and the multilayer ring structure represents the invaginated intestinal segment. In some cases, ultrasonography can show a suborbicular nested mass with a clear boundary, low blood supply, and strong ultrasonic echo. These findings may indicate a lipoma. However, ultrasonography is generally limited to the demonstration of intestinal dilation and obstruction. CT is the most valuable diagnostic method for intestinal intussusception, and it can clearly reveal the typical characteristics of uniform tumor density, a clear border, no reinforcement, and fat (negative) density, allowing for a definite diagnosis [5]. Colonoscopy is also valuable in the diagnosis of intestinal lipoma. Microscopically, a lipoma is characterized as follows: i) it presents as an orange, smooth, submucosal mass protruding into the intestinal lumen with or without a pedicle; ii) the mass can immediately recover from local compression using biopsy forceps (the so-called “cushion sign”); and iii) after repeated biopsy of a certain part of the tumor, the submucosal adipose tissue is visible (termed “naked fat”) [6]. However, endoscopy is of little significance for the diagnosis of intestinal lipoma.

We searched the PubMed database to gain a deeper understanding of lipomas in the small intestine. The following search terms were used: ((lipoma [MeSH Terms]) OR lipoma) AND ((intestine, small [MeSH Terms]) OR small intestine OR small bowel) AND (English [Language]) AND (intussusception [MeSH Terms]). Only 51 eligible articles were retrieved. We excluded articles that described secondary cases or the imaging characteristics of lipomas in the small intestine and only 27 articles remained [7-33] (Table 1).

**Table 1:** Characteristics of reported cases of adult intussusception induced by a lipoma

**Case**	**Sex**	**Age (years)**	**Presentation**	**Diagnostic modality**	**Diagnosis**	**Tumor location**	**Size (cm)**	**Treatment**	**Follow**-**up**	**Authors**
1	Female	29	Abdominal pain	US, CT	Angiolipoma	Ileum	4.3 × 2.5	Exploratory laparotomy	Uneventful	Esnakula K et al. [7]
2	Male	37	Abdominal pain, constipation, and rectal prolapse	Colonoscopy, CT	Colonic lipoma	Colon	5.5	Resection	Well	Pezzolla A et al. [8]
3	Male	75	Rectal bleeding and anemia	Colonoscopy	Lipoma	Ileocecal valve	2.7 × 2.6 × 2.2	Endoscopic resection	Well	Pezzolla A et al. [8]
4	Female	51	Abdominal pain, constipation, and rectal prolapse	Colonoscopy	Lipoma	Sigmoid colon	10.0	Mini-laparotomy	Well	Pezzolla A et al. [8]
5	Male	73	Abdominal pain, nausea, vomiting, and hematochezia	Video capsule endoscopy, CT	Lipoma	Mid-jejunum	2.1	Resection	Well	Lucas LC et al. [9]
6	Female	43	Nausea and upper abdominal pain	MDCT, US	Submucosal lipoma	Small intestine	2.4 × 2.0 × 2.0	Laparotomy	Uneventful	Ako E et al. [10]
7	Female	26	Abdominal pain and vomiting	Plain abdominal film, CT	Ectopic pancreas with abundant fatty infiltration	Small bowel	No exact data	Laparotomy	Unknown	Chuang MT et al. [11]
8	Male	68	Abdominal bloating	DBE	Multiple submucosal lipomas	Jejunum	From 5.5 × 6.0 to 8.5 × 7.5	Resection	Well	Wan XY et al. [12]
9	Male	78	Constipation, nausea, and vomiting	Plain abdominal film, CT	Submucosal lipoma	Ileum	4.0	Resection	Well	Di Saverio S et al. [13]
10	Male	45	Abdominal pain, nausea, and vomiting	CT	Lipoma	Terminal ileum	2.5	Laparoscopy and resection	Well	Whitfield JD et al. [14]
11	Female	62	Abdominal pain, alternating bowel habits, and weight loss	US	Submucosal lipoma	Ileocecal valve	7.0 × 3.0 × 2.5	Laparotomy and right hemicolectomy	Well	Kuzmich S et al. [15]
12	Male	36	Epigastric pain, nausea, and vomiting	X-ray, US, CT	Ileal lipoma	Ileum	2.7 × 2.7 × 4.0	Resection	Well	Akagi I et al. [16]
13	Female	47	Nausea, vomiting, and abdominal pain	CT	Submucosal lipoma	Small intestine	3.0 × 3.0	Laparoscopy and resection	Well	Lin MW et al. [17]
14	Male	54	Acute abdominal pain, nausea, and vomiting	Colonoscopy, X-ray, CT	Lipoma	Ileum	No exact data	Laparoscopy and resection	Well	Oyen TL et al. [18]
15	Female	65	Abdominal cramps and hematochezia	US, CT	Submucosal lipoma	Ileocecal	No exact data	Laparotomy and right hemicolectomy	Well	Rathore MA [19]
16	Male	63	Mid abdominal pain, flatus, and nausea	CT	Lipoma	Ileocecal valve	No exact data	Laparoscopy	Uneventful	McKay R [20]
17	Male	28	Cramps and abdominal pain, vomiting, and diarrhea	X-ray, CT	Meckel’s diverticulum lipoma	Jejunum	3.0	Laparotomy	Uneventful	Ahmed HU et al. [21]
18	Female	20	Cramps and abdominal pain	CT, CECT	Sub mucosal tumor	Small intestine	1.8	Laparotomy	Uneventful	Zissin R [22]
19	Male	40	Cramps and abdominal pain, nausea, and vomiting	CT, MRI, small intestinal barium X-ray	Sub mucosal lipoma	Small bowel	10.0 × 3.0 × 2.0	Laparotomy and reduction	Uneventful	Marino F et al. [23]
20	Male	55	Colicky epigastric pain, nausea, and abdominal distention	X-ray, US, CT	Lipoma	Terminal ileum	No exact data	Laparotomy	Well	Meshikhes AW et al. [24]
21	Male	72	Lack of appetite, early satiety, and nausea	US, CT	Lipoma	Jejunum	10.0 × 5.0	Laparotomy	Uneventful	Mouës CM et al. [25]
22	Male	59	Lower abdominal pain	X-ray, US, colonoscopy, EUS	Lipoma	Ileum	2.7 × 1.9 × 1.9	Laparotomy	Unknown	Watanabe F et al. [26]
23	Female	80	Colicky upper abdominal pain	CT	Lipoma	Jejunum	No exact data	Resection	Well	Ross GJ et al. [27]
24	Female	68	Periumbilical colicky pain, nausea, and vomiting	X-ray, US, CT	Submucosal lipoma	Jejunum	3.5	Resection	Uneventful	Urbano J et al. [28]
25	Male	43	Crampy right upper quadrant pain	X-ray, CT	Multiple submucosal lipomas	Small bowel	1.0–4.0	Laparotomy	Repeat laparotomy due to necrotic bowel	Gates LK Jr et al. [29]
26	Male	12	Cough, vomiting, and intermittent epigastric pain	US, ERCP, barium meal, CT	Submucosal lipoma	Duodenal	10.0 × 6.0 × 4.0	Laparotomy	Unknown	McGrath FP et al. [30]
27	Male	32	Colicky abdominal pain	X-ray, barium meal	Lipoma	Small intestine	8.0 × 5.0	Conservative	Well	Misra SP et al. [31]
28	Female	42	Colicky periumbilical and right upper quadrant pain	Barium enema, CT	Lipoma	Ileocecal valve	3.0	Laparotomy	Unknown	Donovan AT et al. [32]
29	Male	60	Melena	Barium enema	Lipoma	Terminal ileum	2.0 × 3.0 × 4.0	Resection	Unknown	Schnur MJ et al. [33]
30	Female	40	Rectal bleeding	Barium enema	Submucosal lipoma	Terminal ileum	6.0	Resection	Unknown	Schnur MJ et al. [33]
31	Male	65	Small bowel obstruction	Barium enema	Malignant carcinoid tumor	Terminal ileum	0.5 × 1.5 × 3.0	Resection	Unknown	Schnur MJ et al. [33]
32	Male	75	Cramp and abdominal pain	Barium enema	Lipoma	Terminal ileum	5.0	None	Unknown	Schnur MJ et al. [33]
33	Female	74	Rectal bleeding	Barium enema	Lipoma	Terminal ileum	1.0	None	Well	Schnur MJ et al. [33]

Abbreviations: *CT* Computed tomography, *CECT* Contrast-enhanced computed tomography, *MDCT* Multidetector row computed tomography, *MRI* Magnetic resonance imaging, *US* Ultrasonography, *EUS* Endoscopic ultrasonography, *ERCP* Endoscopic retrograde cholangiopancreatography, *DBE* Double-balloon endoscopy.

According to the literature, most lipomas are asymptomatic. They may present as intestinal obstruction or hemorrhage [34]. The lack of specific clinical manifestations makes small intestinal lipomas difficult to diagnose. Their usual location in the small intestine is the ileum. The peak age of incidence is the sixth to seventh decade of life. Despite the development of imaging techniques, only 32% to 50% of cases are diagnosed preoperatively [35]. Intestinal lipomas >2 cm in diameter can cause intussusception. The typical triad of abdominal pain, a sausage-shaped mass, and red jelly stools is seen in children, but rarely in adults. Adult intussusception is a very rare condition, accounting for only around 5% of all intussusception cases and 1% of adult intestinal obstructions [36]. If a lipoma is <2 cm in diameter, it can be resected via endoscopy. However, this method is risky for lipomas with no pedicle. Because fat is a poor conductor, it does not readily solidify after electric coagulation. Bleeding and deep tissue damage may occur. The electric current accumulation during coagulation greatly contributes to the risk of intestinal perforation. Therefore, local intestinal resection is the optimal treatment method. When a lipoma causes intussusception, the intestinal tract should be intraoperatively reset and the local intestine then resected. Otherwise, intestinal resection with anastomosis is feasible. If the condition cannot be diagnosed preoperatively, intraoperative pathological examination helps to determine the best surgical treatment method. In addition, some colonic lipomas are accompanied by colorectal cancer. The intestinal tract should be thoroughly explored during surgery to prevent misdiagnosis.

Definitive surgical resection remains the recommended treatment for adult intussusceptions due to the large proportion of structural causes and the relatively high incidence of malignancy. However, the most optimal surgical management technique remains controversial. Some investigators have stated that small bowel intussusceptions should be reduced only in patients in whom a definitive benign diagnosis has been made preoperatively and should be avoided in patients in whom resection may result in short-bowel syndrome [35]. All surgeons agree that intestinal resection is optimal if there are signs of irreversible bowel ischemia, inflammation, or suspected malignancy [37]. Otherwise, reduction is appropriate, especially when the small bowel is affected, because a considerable length of the bowel can be preserved, thereby preventing the development of short-bowel syndrome.

## Conclusions

In summary, the treatment of intestinal lipoma depends on the clinical manifestations and the size of the tumor. Whether small asymptomatic lipomas need further treatment remains controversial. For symptomatic lipomas, operative resection can be performed to histopathologically exclude liposarcoma and malignant lesions. Laparoscopic operative resection is superior to the traditional open operation. In the presence of complications such as acute intestinal obstruction, intussusception, or massive hemorrhage, operative treatment is recommended. In patients with intestinal ischemia, necrosis, or suspected malignancy, intestinal resection and anastomosis is feasible. Otherwise, reduction is appropriate and prevents the development of short-bowel syndrome.

In this paper, we have described a case involving a lipoma in the terminal ileum. Ultrasonographic and whole-abdomen CT examination showed that the lipoma had caused an ileocolic intussusception and incomplete intestinal obstruction. After the onset of the ileocecal intussusception, the site slowly expanded toward the ascending colon, transverse colon, and splenic flexure of the colon. The lipoma finally fell to the rectum, and intussusception resolved spontaneously.

## Consent

Written informed consent was obtained from the patient for publication of this case and for the accompanying images.

## Abbreviations

CECT: Contrast-enhanced computed tomography; CT: Computed tomography; DBE: Double-balloon endoscopy; ERCP: Endoscopic retrograde cholangiopancreatography; EUS: Endoscopic ultrasonography; MDCT: Multidetector row computed tomography; MRI: Magnetic resonance imaging; US: Ultrasonography.

## Competing interests

All authors have made substantive contributions to the study, and are in agreement with the conclusions of the study. Furthermore, there are no financial competing interests.

## Authors’ contributions

BK and QZ searched the database, selected the articles, and wrote the manuscript. FM supervised the writing of the manuscript. QN, LH, and WC managed the figures. DS supervised the methodology, the selection of the articles, and the writing of the manuscript and is the corresponding author of the paper. All authors have read and approved the final manuscript.

## Authors’ information

The English in this document has been checked by at least two professional editors, both native speakers of English. For a certificate, please see: http://www.textcheck.com/certificate/p5Hkrp.
